# Exploring collagen fibrillogenesis at the nanoscale: Tip‐enhanced Raman imaging of protofibrils

**DOI:** 10.1111/jmi.70029

**Published:** 2025-08-29

**Authors:** Maria A. Paularie, Emerson A. Fonseca, Vitor Monken, André G. Pereira, Rafael P. Vieira, Ado Jorio

**Affiliations:** ^1^ Departamento de Física, Instituto de Ciências Exatas (ICEx) Universidade Federal de Minas Gerais (UFMG) Belo Horizonte Brasil; ^2^ Programa de Pós Graduação em Inovação Tecnológica Universidade Federal de Minas Gerais (UFMG) Belo Horizonte Brasil; ^3^ FabNS, Parque Tecnológico de Belo Horizonte‐BHTec Belo Horizonte Brazil; ^4^ Departamento de Bioquímica e Imunologia, Instituto de Ciências Biológicas (ICB) Universidade Federal de Minas Gerais (UFMG) Belo Horizonte Brasil

**Keywords:** collagen protofibrils, fibrillogenesis, fingerprint, TERS

## Abstract

Collagen, a key structural component of the extracellular matrix, assembles through a hierarchical process of fibrillogenesis. Despite extensive studies on mature collagen fibrils, intermediates such as protofibrils remain underexplored, particularly at the nanoscale. This study presents hyperspectral tip‐enhanced Raman spectroscopy (TERS) imaging of collagen protofibrils, offering chemical and structural insights into early fibrillogenesis by acquiring nanoscale molecular profiles of collagen intermediates. TERS spectra, complemented by atomic force microscopy (AFM) images, reveal characteristic molecular vibrational modes, including the phenylalanine ring breathing mode, amide II and ${\mathrm{CH}}_{2}$/${\mathrm{CH}}_{3}$ stretching vibrations, with distinct spectral signatures compared to mature fibrils.

## 1 INTRODUCTION

**FIGURE 1:**
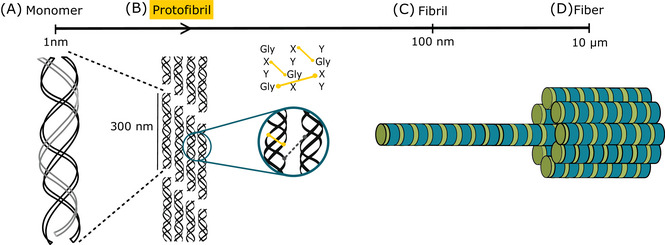
Hierarchical assembly of collagen fibrils through fibrillogenesis. (A) Monomeric tropocollagen units, characterised by a Gly‐X‐Y repeating motif, undergo lateral association into (B) protofibrillar intermediates via enzymatically catalysed cross‐linking. (C) Maturation of protofibrils leads to fibril formation, with highly organised molecular packing. (D) The final supramolecular fiber network emerges, imparting the extracellular matrix with structural integrity, mechanical resilience, and biomechanical functionality.

Collagen consists of three left‐handed polyproline II (PPII) polypeptide chains that arrange into a parallel right‐handed triple helix.^1^, ^2^, ^3^ This architecture arises from its characteristic glycine‐proline‐hydroxyproline (Gly‐X‐Y) repeating motif,^4^ which facilitates structural stabilisation through a combination of interstrand hydrogen bonding, electrostatic interactions, and periodic hydrophobic packing mediated by glycine residues positioned every third residue.^5^, ^6^ The hierarchical assembly of collagen fibrils is initiated by enzymatically catalysed cross‐linking reactions, forming covalent intermolecular bonds that propagate the self‐organisation process.^7^, ^8^ This fibrillogenesis pathway facilitates the lateral association of monomeric tropocollagen units, driving the formation of aggregated intermediates, commonly known as protofibrils, as seen in Figure 1, with diameters ranging up to 100 nm.^9^ As fibrillogenesis progresses, collagen fibrils undergo a stepwise maturation process, culminating in the establishment of densely packed, supramolecular fiber networks. These hierarchical structures provide critical biomechanical properties to the extracellular matrix (ECM), imparting tensile strength, elasticity, and structural integrity essential for tissue architecture and function.^10^, ^11^, ^12^

Intermediates of collagen fibrillogenesis at the nanoscale are inherently challenging to access due to their ensemble dimensions and the requirement for a precisely controlled fibrillogenesis process. Conventional nanoscale biochemical characterisation techniques often present a trade‐off: while some provide exceptional topographical resolution, they lack molecular or spectroscopic insights, whereas others rely on sample labelling,^13^, ^14^ potentially altering native structures, molecular dynamics, or kinetic properties. Tip‐enhanced Raman spectroscopy (TERS) overcomes these limitations by combining the subdiffraction spatial resolution (10–15 nm^15^) of scanning probe microscopy (SPM) with the molecular vibrational specificity of Raman spectroscopy.^16^ This approach enables label‐free molecular profiling of weakly scattering biological molecules,^17^ providing unique means to investigate transient and structurally heterogeneous protein assemblies that remain inaccessible through traditional imaging techniques.

Collagen has been extensively characterised using advanced spectroscopic methods, including confocal Raman,^18^ FT‐Raman,^19^ FTIR,^20^ coherent anti‐Stokes Raman scattering (CARS),^21^ and stimulated Raman scattering (SRS).^22^ However, despite these advances, hyperspectral tip‐enhanced Raman spectroscopy (TERS) studies specifically addressing collagen intermediates such as protofibrils remain scarce, with only a few reports in the literature.^23^ This study presents spatially resolved TERS images of collagen protofibrils, leveraging some biomarker‐specific Raman band signals to achieve nanoscale resolution, while simultaneously acquiring atomic force microscopy images. By extending beyond traditional single‐point TERS analyses of biological samples, this study provides TERS images of collagen intermediates based on the phenylalanine ring breathing mode band, amide II mode and ${\mathrm{CH}}_{2}$/${\mathrm{CH}}_{3}$ stretching vibrations.

As previously discussed by Zenobi et al.,^24^, ^25^, ^26^ most TERS studies on biological samples rely on single‐point measurements or limited line scans. However, such discrete spectral acquisitions often yield inconsistent Raman fingerprints, limiting the reliability of molecular assignments. In contrast, hyperspectral TERS imaging circumvents these inconsistencies by providing spatially resolved spectral maps with nanoscale precision, enabling the correlation of molecular composition with topographical features. This study demonstrates that a robust and reproducible TERS fingerprint can be achieved.

## 2 EXPERIMENTAL SECTION

### 2.1 Sample preparation and Topography characterisation

Bovine Achilles tendon Type I collagen (Sigma, St. Louis, MO) was solubilised in 1 M acetic acid (pH 2) at 4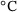 to 1 mg/mL, then diluted with phosphate‐buffered saline (PBS; 137 mM NaCl, 2.7 mM KCl, 10 mM Na_2_HPO_4_, 1.8 mM KH_2_PO_4_, pH 7.4) to 0.167 mg/mL, and pH adjusted to 7.4. Fibril assembly was interrupted by diluting the solution into distilled water (1:5) and stored at 4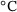. For single‐molecule imaging, 20 μL of the sample suspension was deposited onto a clean coverglass (Fisher Scientific, Pittsburgh, PA) and incubated for 10 min in a humidified chamber to prevent solvent evaporation and maintain a stable droplet concentration. The chamber was kept at ambient temperature to ensure consistent fibril deposition and minimise uncontrolled aggregation. The sample was rinsed, first with PBS, and then with ultra‐high‐quality water (UHQ) to deter salt crystal formation. Finally, the sample was dried using a gentle stream of dry N_2_ before TERS experiments.

Preliminary AFM experiments were performed using a NaioAFM system (Nanosurf, Liestal), equipped with a Tap190‐G probe (Budget Sensors). Scanning parameters were set to a line acquisition time of 2 s, with 512 lines per scan, a setpoint of 50%, and an amplitude of 200 mV. The samples were imaged in dry conditions in tapping mode. Multiple AFM tests were performed to ensure the protofibrils were within the expected size range. Cross‐sectional analysis of 20 protofibrils reveals that the nanotape heights are 53.2±15.6nm and the widths are 65±31nm. The AFM images also demonstrate that the protofibrils tend to stand close, likely due to hydrophobic interactions and electrostatic forces between their surfaces. This data validated the reliability of the preparation method and the suitability of the sample dimension for subsequent TERS characterisation.

### 2.2 Raman experimental setup

**FIGURE 2:**
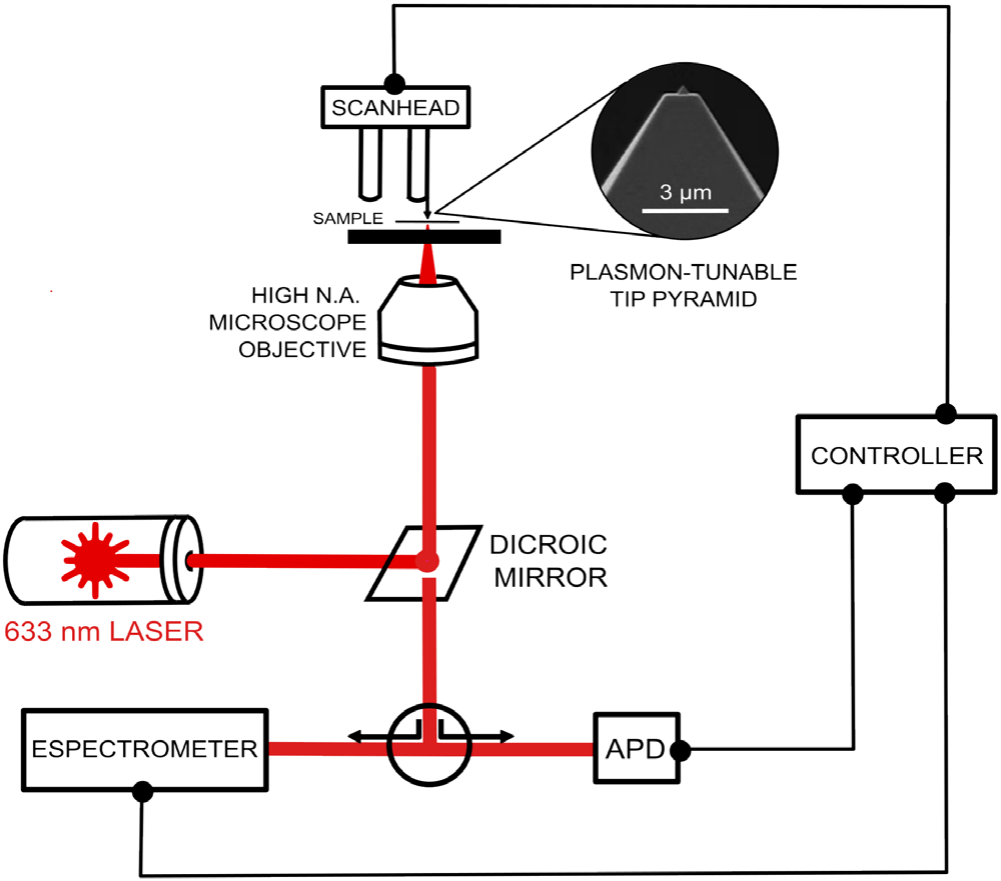
Schematic illustration of the TERS setup.^27^ The system operates in a back‐scattering geometry, with a high NA objective lens focusing a λ = 632.8  nm laser onto the sample. The AFM scanning head, equipped with a gold plasmon‐tunable tip pyramid (PTTP),^27^, ^28^, ^30^ is positioned above the sample.

The equipment utilised for nano‐Raman measurements was the Porto system prototype,^27^ as seen on Figure 2. This setup operates in a back‐scattering geometry, combining confocal Raman spectroscopy with AFM to achieve nanometric resolution and spectral mapping. A radially polarised HeNe laser (λ = 632.8  nm, 748 μW) is focused onto the sample using an oil‐immersion objective lens (NA = 1.4). The AFM scanning head is equipped with a eletrochemically etched gold plasmon‐tunable tip pyramid (PTTP).^28^, ^29^

**FIGURE 3:**
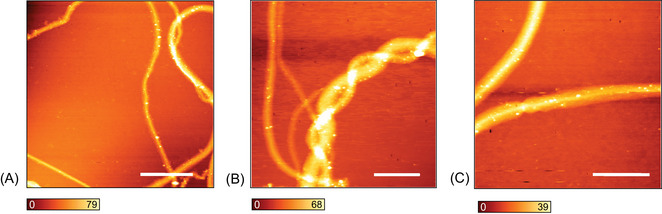
Representative AFM height images of collagen protofibrils. The protofibrils vary in size but stay consistent below 100 nm. (A–C) The morphology of the protofibrils at different magnifications. (A) Scale bar 15 μm. (B) Scale bar 8 μm. (C) Scale bar 5 μm.

Measurements were conducted over a 1 μm 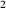 area, divided into 32 pixels, with each pixel having a 5‐s acquisition time. After acquiring the TERS hyperspectral map, the probe was immediately retracted, and a second hyperspectral map was recorded under the same experimental parameters to obtain the corresponding far‐field Raman hyperspectral data. The AFM topography data was processed using Gwyddion software (v2.62), while Raman spectra and TERS intensity maps were analysed with PortoFlow software (v1.18).

For data processing, PCA was performed, retaining the first 10 principal components for data dimensionality reduction and reconstructing the data using the top 5 components to enhance signal‐to‐noise ratio while preserving relevant spectral features, being extremely careful not to create spurious bands.

For comparison with the TERS measurements, confocal Raman Spectroscopy was performed using a Raman Microscope WITec Alpha 300 SAR, with a λ=632.8nm excitation laser. To obtain bulk spectra, larger fibrils were prepared through higher incubation time (1 h) to ensure optical detectability. A 50× (0.55 NA) objective was employed, and spectra were integrated for 5 × 5 s with 5 mW laser power.

## 3 RESULTS AND DISCUSSION

**FIGURE 4:**
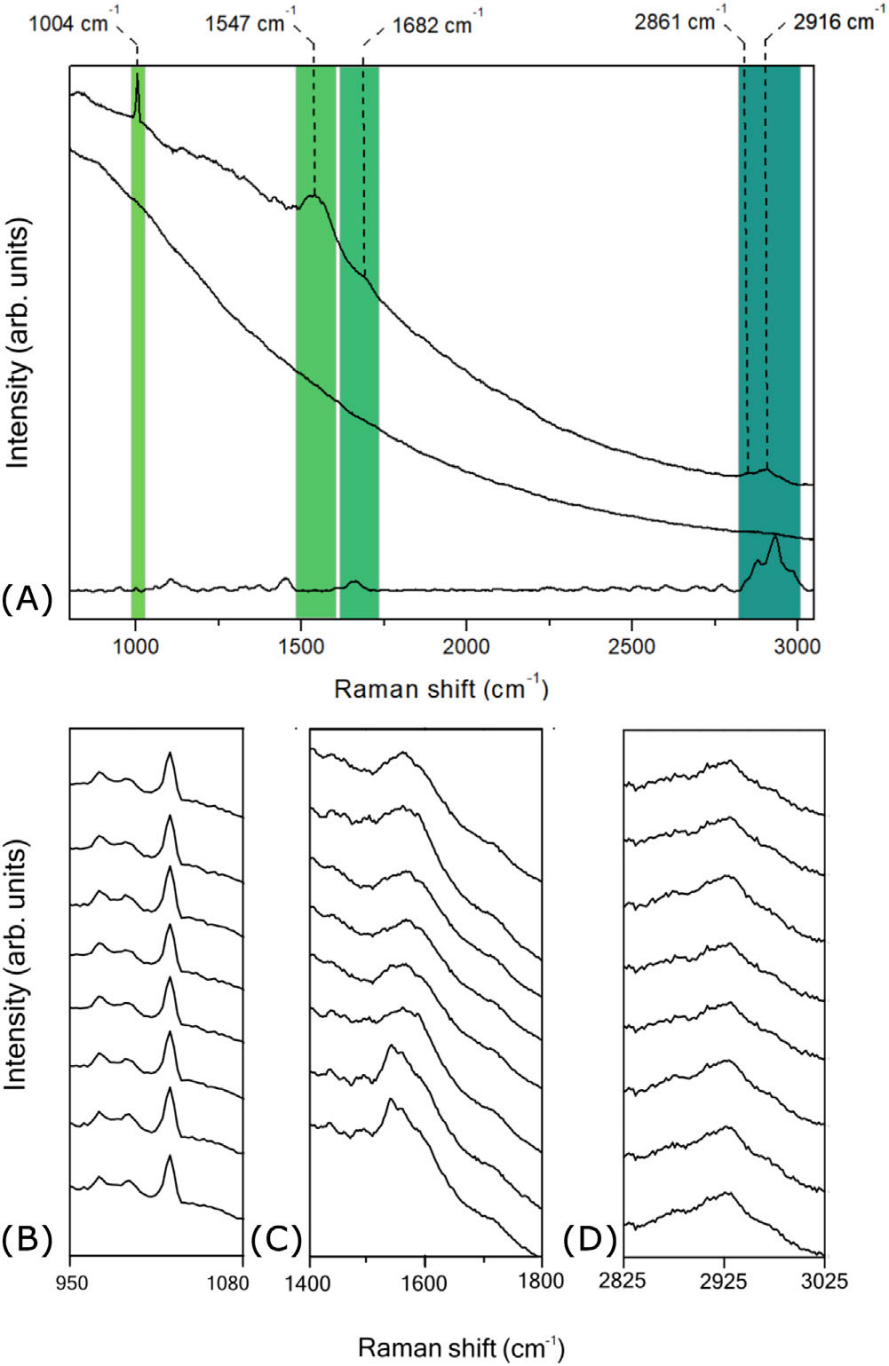
TERS spectrum extracted from the hyperspectral map on fibril (top), compared with control (TERS‐off fibril) and bulk confocal Raman spectra (bottom). The phenylalanine ring breathing mode (1004 ${\mathrm{cm}}^{-1}$), amide II (1547 ${\mathrm{cm}}^{-1}$), amide I (1682 ${\mathrm{cm}}^{-1}$) and ${\mathrm{CH}}_{2}$/${\mathrm{CH}}_{3}$ stretching vibrations (2850–2930 ${\mathrm{cm}}^{-1}$) are highlighted by the green hashed bars and their spatial distribution will be shown in Figure 5. Panels b–d display these highlighted vibrational modes along the fibril during map acquisition: (b) phenylalanine; (c) amide II and I; and (d) ${\mathrm{CH}}_{2}$/${\mathrm{CH}}_{3}$ stretching, displayed in acquisition order from bottom (first) to top (last). One spectrum was collected every four pixels (eight in total).

According to the AFM images, the protofibrils exhibit a tubular‐like morphology and are remarkably long, as seen on Figure 3, making it challenging to identify their termini. Their dimensions vary considerably. Even with high acquisition times per line and a large number of scan lines, the resolution did not reveal a well‐defined D‐periodic banding pattern, further supporting the characteristic features of collagen intermediates.^31^ As previously discussed, though collagen has been extensively characterised using a plethora of spectroscopic techniques, these approaches have primarily focused on mature fibrils. For comparison with the TERS data, a confocal Raman spectrum of a bulk fibril sample was collected. The observed spectral band pattern is consistent with those reported in the literature: phenylalanine aromatic ring breathing mode at 1004 ${\mathrm{cm}}^{-1}$, scissoring modes at 1447 ${\mathrm{cm}}^{-1}$, amide I mode at 1661 ${\mathrm{cm}}^{-1}$, and in higher frequency Raman shifts, the ${\mathrm{CH}}_{2}$ stretching (∼2854 ${\mathrm{cm}}^{-1}$) and ${\mathrm{CH}}_{3}$ (∼2930 ${\mathrm{cm}}^{-1}$) bands. In Figure 4, the confocal Raman spectrum is compared with representative spectra from the hyperspectral TERS map of a protofibril region (TERS‐on fibril) and a non‐protofibril region (TERS‐off fibril). All spectra were normalised to the highest intensity of the TERS‐on fibril spectrum, with the bulk Raman spectrum further scaled by a factor of 4 for improved visualisation. Note that the TERS spectrum exhibits a broad background signal, in contrast to the stable baseline observed in the confocal Raman spectrum and whose origin is likely the photoluminesence of the rough metal surface of the gold PTTP probe. Notably, none of the spectra shown have undergone background correction. Table 1 presents a comparative analysis of the experimental TERS and confocal Raman spectroscopy peak positions, along with their corresponding band assignments and references. Interestingly, the amide II band is absent in the confocal Raman spectrum but appears in the TERS spectrum, phenomena observed by Refs. 32–35, showing that vibrational modes such as amide II are inherently weak in the bulk protein, making them difficult to detect in confocal Raman measurements, but become pronounced in the enhanced spectra. Additionally, an expressive blueshift was observed for the amide I band, from 1660 ${\mathrm{cm}}^{-1}$ in the confocal spectrum to 1682 ${\mathrm{cm}}^{-1}$ in the TERS spectrum. It has been documented that vibrational mode positions can change in enhanced Raman spectra relative to confocal spectra depending on the interaction of the molecule with the metal probe,^32^, ^36^, ^37^ and substantial shifts are not uncommon. For instance, Blum et al.^38^ reported a shift of 16 ${\mathrm{cm}}^{-1}$ in the TERS spectrum relative to the corresponding confocal Raman spectrum, highlighting the importance of directly comparing TERS and confocal measurements to accurately identify vibrational bands.

**TABLE 1:** Comparison of experimental TERS and confocal Raman spectroscopy of discussed peak positions with their corresponding band assignments and references.

Confocal Raman (${\mathrm{cm}}^{-1}$)	TERS (${\mathrm{cm}}^{-1}$)	Band assignment	References
1000	1004	phenylalanine	39, 40
1660	1682	amide I	41, 42
—	1547	amide II	43, 44
2870	2861	${\mathrm{CH}}_{2}$	44, 45
2932	2916	${\mathrm{CH}}_{3}$	44, 45

**FIGURE 5:**
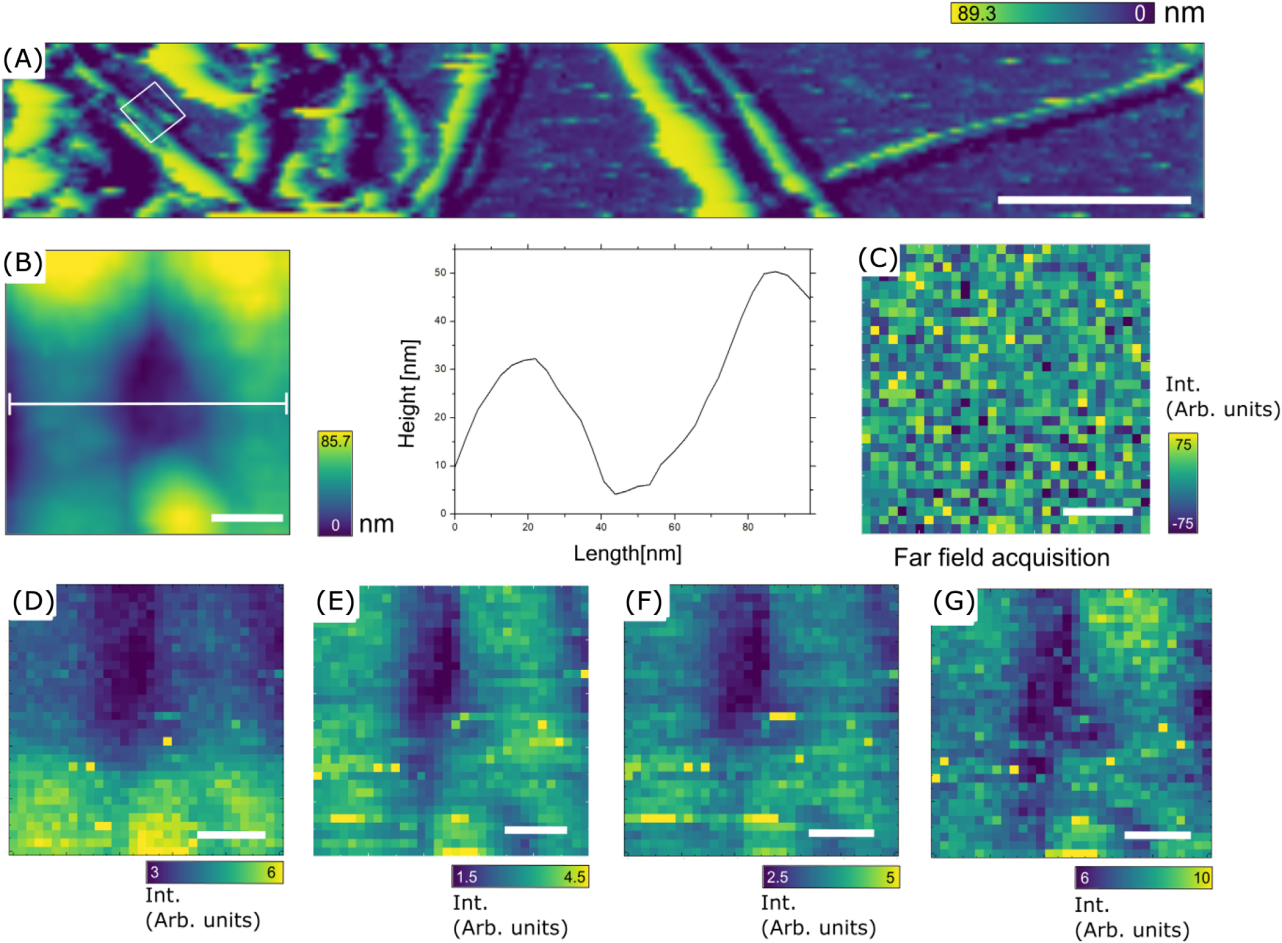
(A) AFM image showing the spatial distribution of collagen protofibrils. The highlighted square marks the region specifically chosen for subsequent measurements. Scale 5 bar μm. (B) High‐resolution AFM image of selected region acquired with the TERS equipment with the line profile on the right, revealing two fibrils: one measuring approximately 30 nm in diameter and the other, 50 nm (see line profile on the right). The smaller fibril appears to partially overlap the larger fibril. (C) Far‐field acquisition of the same region. (D–G) TERS experiments at the same location of (B), scale bars 250 nm. (D) ${\mathrm{CH}}_{2},{\mathrm{CH}}_{3}$ stretching band (2850–2930 ${\mathrm{cm}}^{-1}$); (E) amide I band (1661 ${\mathrm{cm}}^{-1}$); (F) amide II band (1547 ${\mathrm{cm}}^{-1}$); (G) phenylalanine band (1004 ${\mathrm{cm}}^{-1}$).

Figure 5B presents the AFM image acquired concurrently with the TERS maps (Figure 5D–G), providing spatially resolved insights into the nanoscale distribution of bands detected in the hyperspectral data. The selected bands ${\mathrm{CH}}_{2}$, ${\mathrm{CH}}_{3}$ stretching band (2850–2932 ${\mathrm{cm}}^{-1}$), amide I mode (1682 ${\mathrm{cm}}^{-1}$), amide II mode (1547 ${\mathrm{cm}}^{-1}$) and the aromatic ring breathing marker band of phenylalanine (1004 ${\mathrm{cm}}^{-1}$) exhibit a clear dimension correlation with the structural features observed in the AFM measurements of the same region. According to the Nyquist criterion,^46^ they present a spatial resolution of approximately 71.9 nm (32 × 32 pixels over a 1 μm 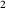 area) and were plotted using integral. Immediately after the measurement, the probe was retracted, and considering the same laser power and acquisition times, the hyperspectral far‐field was acquired on the same region (Figure 5C). No detectable Raman bands were observed under these conditions, confirming that the previously recorded spectral features originated exclusively from the near‐field enhancement in the TERS setup.

The ${\mathrm{CH}}_{2},{\mathrm{CH}}_{3}$ band map exhibits a trend that closely aligns with the regions showing attenuation or enhancement in signal intensity, as seen on Figure 5D. The amide I and amide II band maps demonstrate a more consistent distribution. Interestingly, the map for the aromatic ring breathing marker band of phenylalanine displays a zigzag‐like pattern on the second protofibril, suggesting spatial heterogeneity in the underlying molecular orientation or packing density. Moreover, although several weak bands are present in the 1000–1500 ${\mathrm{cm}}^{-1}$ region in both TERS and confocal Raman spectra, these appear as broadened and poorly defined features in the TERS case, largely obscured by the luminescent background from the Au tip. Given this limitation. For this reason, we decided not to make any analysis based on this spectral region. The assembly pattern characteristic of collagen fibrils could play a role in the observed spatial heterogeneity. Given the 71.9 nm resolution of the TERS maps, it is conceivable that the periodic organisation of collagen molecules within the protofibril could influence the local enhancement or attenuation of vibrational modes under TERS conditions and be seen on hyperspectral data. This scenario is unlikely, since the trend is not followed by the predicted architecture of the D‐period banding packing pattern of 67 nm repetition^47^, ^48^ on the rest of the material.

## 4 CONCLUSIONS

This study presents hyperspectral tip‐enhanced Raman spectroscopy (TERS) imaging of collagen protofibrils, offering a perspective on the molecular and structural characteristics of these intermediates in collagen fibrillogenesis. The spectral band pattern observed is consistent with those reported in the literature, including the phenylalanine ring breathing mode, amide II and ${\mathrm{CH}}_{2}$/${\mathrm{CH}}_{3}$ stretching vibrations. Notably, the amide I mode is less intense in the TERS spectra.

By being able to map the spatial distribution of the key biomarker bands detected with a spatial resolution of 71.9 nm, we were able to achieve high spatial resolution, revealing distinct separation between individual fibrils. This spatial resolution also facilitated the identification of regions within the fibrils, revealing distinct correlation between the molecular features observed in the Raman maps and the structural characteristics measured by AFM, supporting the accuracy of the chemical‐structural information.

The mapping results reveal distinct spatial variations across different vibrational modes, including heterogeneity in the phenylalanine band, where a zigzag‐like pattern was observed, possibly indicative of molecular orientation or packing density variations. While the amide I and II maps show a more uniform distribution, the ${\mathrm{CH}}_{2}$/${\mathrm{CH}}_{3}$ stretching band displayed a clear trend of signal intensity fluctuations. These fluctuations are consistent with photochemical degradation, as suggested by the reduced intensity of certain spectral features and the diminished resemblance to bulk Raman spectra of the same molecule.^49^, ^50^, ^51^, ^52^ In particular, the amide I band (a reliable Raman marker of peptide bonds) is attenuated and appears only as a smaller feature adjacent to the amide II band. This behavior is in line with previous reports of photoinduced sample degradation under laser irradiation, where plasmon‐enhanced processes can induce bond cleavage in the peptide backbone.^32^, ^38^, ^53^

The results show that TERS, when combined with AFM, provides a powerful platform for the investigation of weakly scattering biological molecules, such as collagen protofibrils, without the need for labelling. The hyperspectral TERS maps demonstrated that key vibrational modes, including the phenylalanine ring breathing, amide II, and ${\mathrm{CH}}_{2}$/${\mathrm{CH}}_{3}$ stretching bands, are preserved in protofibrils, revealing biochemical compositional features at nanoscale resolution.
